# Effect of Graphene Oxide and Silver Nanoparticle Hybrid Composite on *Acinetobacter baumannii* Strains, Regarding Antibiotic Resistance and Prevalence of AMP-C Production

**DOI:** 10.3390/medicina59101819

**Published:** 2023-10-12

**Authors:** Povilas Lozovskis, Erika Skrodenienė, Virginija Jankauskaitė, Astra Vitkauskienė

**Affiliations:** 1 Department of Laboratory Medicine, Kaunas Klinikos, The Hospital of Lithuanian University of Health Sciences, 50161 Kaunas, Lithuania; erika.skrodeniene@lsmu.lt (E.S.); astra.vitkauskiene@lsmu.lt (A.V.); 2 Department of Production Engineering, Kaunas University of Technology, 51424 Kaunas, Lithuania; virginija.jankauskaite@ktu.lt

**Keywords:** graphene, graphene oxide, graphene nanoparticles, silver, *A. baumannii*, antimicrobials

## Abstract

*Background and Objectives:* Growing antibiotic resistance among bacteria is a global issue that is becoming harder and more expensive to solve. Traditional treatment options are becoming less effective, causing more fatal outcomes of nosocomial infections. Since the development of new antibiotics has stagnated in the last decade, a novel approach is needed. *Materials and Methods:* Graphene-based materials are being developed and tested for various applications, and the medical field is no exception. We tested 98 clinical *A. baumannii* strains for antibiotic resistance, AMP-C production and the effectiveness of a graphene oxide and silver nanoparticle hybrid nanocomposite. The disc diffusion method was used to determine antibiotic susceptibility results. Antibiotic discs containing cefotaxime, cloxacillin and clavulanate were used to detect AMP-C production. The effectiveness of the GO–Ag hybrid nanocomposite was determined by counting colony forming units (CFUs) after a suspension of *A. baumannii* and the GO–Ag hybrid nanocomposite was plated on MH agar and incubated overnight to grow colonies. *Results*: In our research, we found that *A. baumannii* strains are resistant to the majority of commonly used antibiotics. Antibiotic resistance levels and AMP-C production can be factors, indicating the better effectiveness of the graphene oxide and silver nanoparticle hybrid nanocomposite. *Conclusions*: In this study, a GO–Ag hybrid nanocomposite was shown to have the potential to fight even the most problematic bacteria like *A. baumannii*.

## 1. Introduction

The last few years have brought many challenges to the health care system worldwide. This has resulted in the increased use of various drugs, including antibiotics [1]. The dependence between the inadequate usage of antibiotics and emerging resistance has been known for a long time [2], but efforts to reduce the unneeded use of antibiotics appear to be futile, since global use of antibiotics is ever-growing [3,4,5]. Recent increases in use of disinfectants may have led to increased evolutionary pressure for pathogens to adapt new resistance mechanisms that will be effective against currently used antimicrobial drugs [6]. We will need a novel approach to prevent and treat new and more resistant bacterial strains. Graphene-based materials have already demonstrated their antimicrobial properties against various pathogens [7,8]. There are few suggested ways to facilitate interaction between bacteria and graphene-based nanoparticles. The synergy of different antimicrobial activities seems to be the most important factor for the bactericidal effectiveness of the aforementioned materials [9]. Enriching graphene-based materials with silver nanoparticles has been shown to increase their effectiveness against pathogens like *S. aureus*, *E. coli*, *P. aeruginosa* and even *C. albicans* [10]. Our previous studies have shown that a graphene oxide and silver nanoparticle (GO–Ag) hybrid nanocomposite has antimicrobial properties against *P. aeruginosa*. To further test its potential, we investigated the effect of the GO–Ag hybrid nanocomposite on a more environment-resistant bacteria—*Acinetobacter baumannii*.

*A. baumannii*, just a couple of decades ago, was known as a species of soil bacteria causing opportunistic infections, mainly in rural communities. However, with high innate resistance, this bacterium quickly established its presence in health care facilities and became one of the most common dangerous nosocomial pathogens. As long as *A. baumannii* can acquire new resistance mechanisms, therapeutic options are narrowing [11]. This rapid growth in *A. baumannii* resistance to multiple antibiotics was an essential factor prompting the World Health Organization (WHO) to add *A. baumannii* to the group of pathogens that cause concern, known as ESKAPE (*Enterococcus faecium*, *Staphylococcus aureus*, *Klebsiella pneumoniae*, *A. baumannii*, *Pseudomonas aeruginosa* and *Enterobacter* species) pathogens. It is difficult to compare current *A. baumannii* antibiotic resistance levels with historic data, due to changes in taxonomy, but research from the early 2000s show that *A. baumannii* had already developed resistance to virtually all of the then-used antibiotics, and a quarter of all nosocomial infection-causing strains could have been carbapenem-resistant [12]. In a decade, resistance to carbapenems has increased more than twofold [13].

The virulence factors of *A. baumannii* include porins, capsular polysaccharide, lipopolysaccharide, various metal acquisition systems, several types of secretion systems, productions of antibiotic degrading enzymes and others [14]. One of the prevalent mechanisms of antibiotic resistance in *A. baumannii* is AMP-C β-lactamase production [15]. It is associated with resistance to many widely used β-lactam antibiotics.

An excellent way to approach the problem is to prevent nosocomial infections instead of treating them [16]. This could be achieved by coating surfaces of hospital equipment that cannot be disinfected by other means with materials with antimicrobial properties like as GO–Ag hybrid nanocomposites. 

## 2. Materials and Methods

### 2.1. A. baumannii Isolation and Antibiotic Susceptibility Testing

A collection, containing 98 clinical *A. baumannii* strains, was collected from various clinical materials (blood, lower respiratory tract and surgical wounds) from 2015 to 2020 in The Hospital of Lithuanian University of Health Sciences Kaunas Clinic. For our study, samples were chosen randomly, but we ensured that only one sample from different patients was kept for further testing. Strains were identified using MALDI-TOF (Bruker, Billerica, MA, USA) mass spectrometry. Antibiotic susceptibility was determined using the disc diffusion method (Becton, Dickinson and Company, Franklin Lakes, NJ, USA) on freshly cultivated colonies. Susceptibility was determined for 16 antibiotics. *A. baumannii* strains were inoculated in trypticase soy broth with 15% glycerin and kept in a freezer at −80 °C for further testing. For more accessible data management, antibiotics were grouped into ß-lactam (ampicillin-sulbactam, piperacillin-tazobactam, cefoperazone-sulbactam, cefepime, ceftazidime, imipenem, meropenem) and second line (gentamicin, amikacin, tobramycin, ciprofloxacin, doxycycline, tetracycline, trimethoprim-sulfamethoxazole, tigecycline colistin) antibiotics. Inhibition zones around antibiotic discs were evaluated according to EUCAST (European Committee on Antimicrobial Susceptibility Testing) guidelines.

### 2.2. Detection of AMP-C ß-Lactamases

To determine if any of the *A. baumannii* strains were producing AMP-C β-lactamases, we used the ESBL AMPC Detection kit (Abtek Biologicals, Liverpool, UK). Mueller–Hinton agar plates were swabbed with *A. baumannii* culture suspension in saline of 0.5 McFarland turbidity. Four discs (CTX30 (30 µg cefotaxime), CXV40 (30 µg cefotaxime and 10 µg clavulanate), CTC230 (30 µg cefotaxime and 200 µg cloxacillin), CCC240 (30 µg cefotaxime, 10 µg clavulanate and 200 µg cloxacillin) were placed on the agar plates. Plates were incubated at 37 °C for 24 h. The following day, the inhibition zones were measured. Results were interpreted using the manufacturer’s instructions. 

### 2.3. GO–Ag Hybrid Nanocomposite Effect on A. baumannii Strains

Silver nanoparticles, stabilized with polyvinylpyrrolidone (10 mg/mL), were drop-wise added to a graphene oxide dispersion (5 mg/mL) at a ratio of 1.3:1. The detailed preparation of the GO–Ag hybrid nanocomposite was described in our previous studies [17]. To determine the effect of the GO–Ag hybrid nanocomposite on *A. baumannii* strains, we used the method of counting colony forming units (CFUs) after different incubation times with the GO–Ag hybrid nanocomposite. To make 0.5 MF turbidity bacterial suspension in saline, we used *A. baumannii* strains cultivated on 5% blood agar (Becton, Dickinson and Company) overnight at 37 °C in an incubator. Volumes of 180 µL of bacterial suspension and 20 µL of GO–Ag hybrid nanocomposite were mixed in a tube and vortexed. The tubes were placed in a 37 °C incubator. After 45 and 90 min, the samples were spread on Mueller–Hinton agar plates using a 1 µL loop and plating in a streak pattern. Grown *A. baumannii* CFUs were counted after plates were incubated overnight at 37 °C.

Statistical analysis was undertaken using IBM SPSS Statistics 26 (IBM, Armonk, NJ, USA) software. Chi squared tests and Fisher’s exact tests were performed. Results were considered statistically significant if *p* < 0.05.

## 3. Results

### 3.1. Antibiotic Resistance and AMP-C β-lactamases Production

AMP-C production was found in 32 out of 98 (32.7%) *A. baumannii* strains (Table 1). Antibiotic resistance levels, among tested *A. baumannii* strains, were high against the most tested antibiotics (Table 1). 

Almost all tested *A. baumannii* strains were resistant to ciprofloxacin, ceftazidime and piperacillin-tazobactam (98.0%, 96.9% and 95.5%, respectively) (Table 1). Over 90% of strains were resistant to imipenem, cefepime and meropenem (93.9%, 92.9% and 92.9%, respectively). The most effective antibiotic against tested *A. baumannii* strains was colistin, as all strains were susceptible. Among all 98 *A. baumannii* strains that we tested for antibiotic resistance, only one was susceptible to all 16 antibiotics, and one strain was resistant only to tetracycline.

AMP-C-producing strains were significantly less resistant to ampicillin-sulbactam, cefoperazone-sulbactam, aminoglycosides and tigecycline than AMP-C-non-producing strains (Table 1). Only 40.6% and 25.0% of AMP-C-producing *A. baumannii* stains were resistant to ampicillin-sulbactam (*p* < 0.001) and cefoperazone-sulbactam (*p* < 0.001), respectively. In comparison, 93.9% and 84.9% of AMP-C-non-producing strains were resistant to these antibiotics. Resistance to other ß-lactam antibiotics was similar among AMP-C-producing and -non-producing strains. Resistance to gentamicin, amikacin and tobramycin among AMP-C-producing strains was 68.8%, 68.8% and 71.9%, respectively, while AMP-C-non-producing strains were significantly more resistant at 90.9%, 89.4% and 87.9%, respectively (*p* = 0.011, *p* = 0.005, *p* = 0.049). The only significant difference was observed regarding resistance to tigecycline. AMP-C-producing *A. baumannii* strains were less resistant to tigecycline than AMP-C-non-producing strains: 56.3% vs. 77.3%, respectively (*p* = 0.033).

No significant differences between AMP-C-producing and -non-producing *A. baumannii* strains were found, regarding resistance to the remaining antibiotics.

### 3.2. Effect of GO–Ag Hybrid Nanocomposite on AMP-C-Producing and AMP-C-Non-Producing A. baumannii Strains

**Figure 1:**
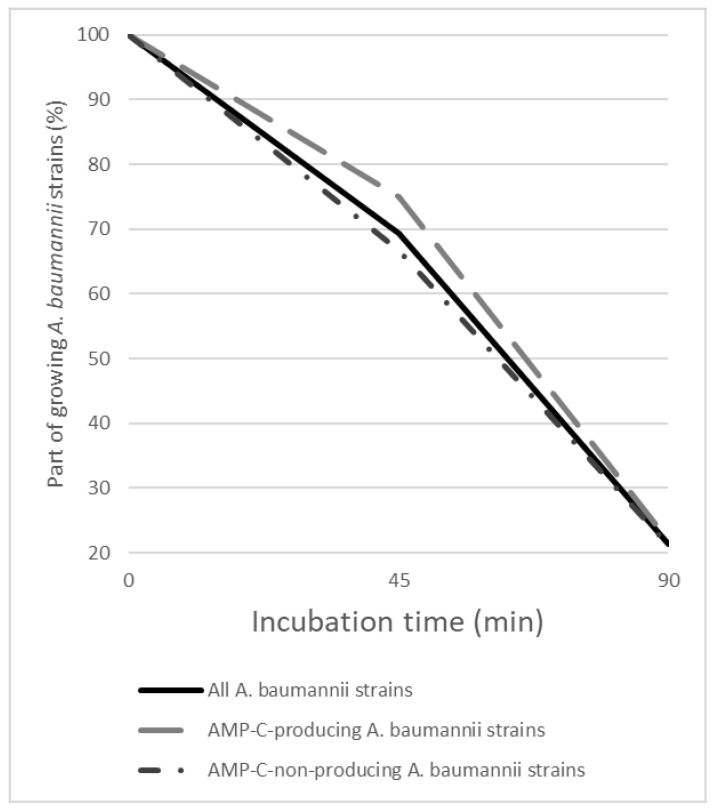
A graph showing reduction in growth of *A. baumannii* strains due to increased incubation with GO–Ag hybrid nanocomposite over time.

The influence of *A. baumannii* and GO–Ag hybrid nanocomposite interaction time was determined by the growth of *A. baumannii* colonies (Figure 1).

After 45 min of incubation with the GO–Ag hybrid nanocomposite, AMP-C-non-producing *A. baumannii* strains were affected slightly more than AMP-C-producing strains. Growth was observed in 75.0% of AMP-C-producing strains and in 66.7% of AMP-C-non-producing strains, but the difference was not statistically significant. When incubation time was increased to 90 min, the difference between AMP-C-non-producing and -producing *A. baumannii* strains was even smaller. Only 21.2% of AMP-C-non-producing and 21.9% of AMP-C-producing strains were growing.

### 3.3. Effect of GO–Ag Hybrid Nanocomposite on Antibiotic-Resistant A. baumannii Strains

**Figure 2:**
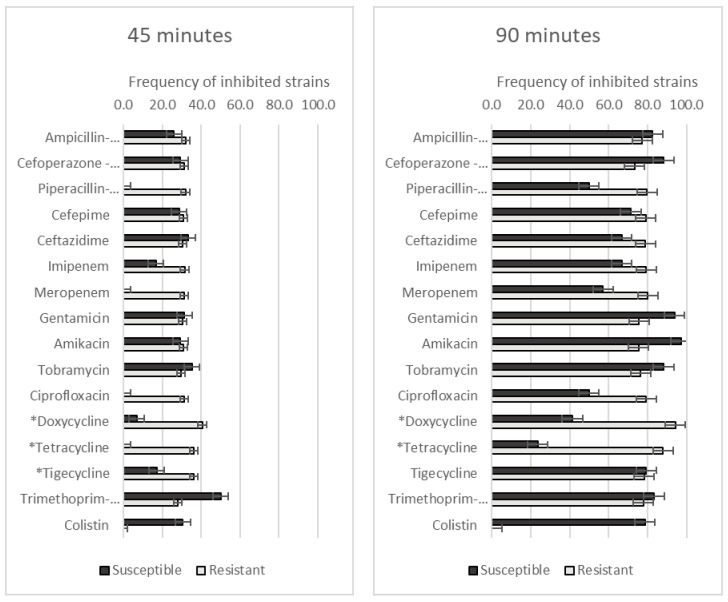
The frequency of *A. baumannii* strains with different antibiotic resistance levels, affected by GO–Ag hybrid nanocomposite after 45 and 90 min of incubation. * *p* < 0.05.

The GO–Ag hybrid nanocomposite had various effects on *A. baumannii* strains with different levels of antibiotic resistance (Figure 2). After 45 min of incubation with the GO–Ag hybrid nanocomposite, *A. baumannii* strains resistant to doxycycline, tetracycline and tigecycline were inhibited significantly more frequently than susceptible strains (40.6% vs. 6.9%, *p* = 0.001; 36.1% vs. 0.0%, *p* = 0.002; 36.2% vs. 17.2%, *p* = 0.049, respectively) (Figure 2). When the incubation time was increased to 90 min, significantly stronger inhibition was observed among doxycycline- and tetracycline-resistant *A. baumannii* strains (94.2% vs. 41.4%, *p* < 0.001; 88.0% vs. 23.7%, *p* < 0.001, respectively).

**Figure 3:**
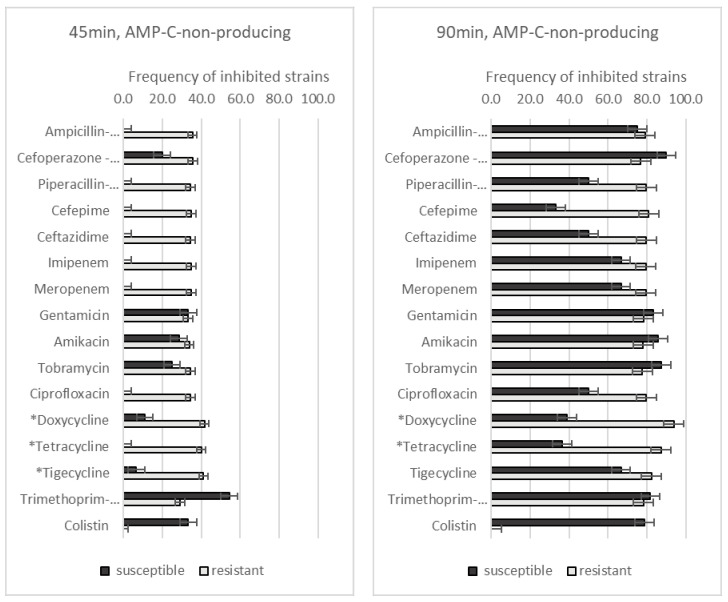
The frequency of AMP-C-non-producing *A. baumannii* strains with different antibiotic resistance levels, effected by GO–Ag hybrid nanocomposite after 45 and 90 min of incubation. * *p* < 0.05.

We also compared the GO–Ag hybrid nanocomposite effect on AMP-C-non-producing (*n* = 66) and -producing (*n* = 32) strains, regarding antibiotic resistance. After 45 min of incubation with the GO–Ag hybrid nanocomposite, AMP-C-non-producing *A. baumannii* strains that were resistant to doxycycline (41.7% vs. 11.1%, *p* = 0.016), tetracycline (40.0% vs. 0.0%, *p* = 0.0027), and tigecycline (41.2% vs. 6.7%, *p* = 0.01) were significantly more affected than susceptible strains (Figure 3). When the incubation time was increased to 90 min, the only significant differences were those regarding doxycycline (93.8% vs. 38.9%, *p* < 0.001) and tetracycline (87.3% vs. 36.4%, *p* = 0.001) resistance.

**Figure 4:**
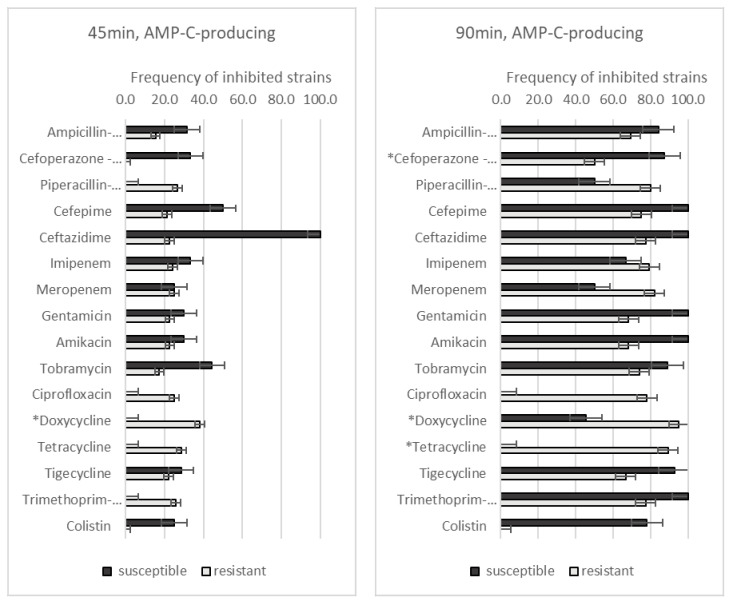
The frequency of AMP-C-producing *A. baumannii* strains with different antibiotic resistance levels, affected by GO–Ag hybrid nanocomposite after 45 and 90 min of incubation. * *p* < 0.05.

When we compared the effect of the GO–Ag hybrid nanocomposite on the AMP-C-producing strains (Figure 4), after 45 min of incubation, a significant difference was only found regarding doxycycline resistance. Resistant *A. baumannii* strains were affected more severely than susceptible strains (38.1% vs. 0.0%, *p* = 0.019). After 90 min of incubation, strains resistant to doxycycline (95.2% vs. 45.5%, *p* = 0.03) and tetracycline (89.3% vs. 0.0%, *p* = 0.001) were affected more than susceptible strains. *A. baumannii* strains susceptible to cefoperazone-sulbactam were affected more severely than resistant strains (87.5% vs. 50.0%, *p* = 0.047).

## 4. Discussion

*A. baumannii* has quickly become a major nosocomial threat. The plethora of its virulence factors and ever-growing resistance to antimicrobials are the main factors that have caused *A. baumannii* to be established among the most problem-causing pathogens in health care facilities [14]. The problem of *A. baumannii* is likely to become even more severe, since the natural habitat of this bacteria is increasing, caused by climate change [18]. In the near future, *A. baumannii* is likely to occupy niches left by bacteria that will not be capable of adapting to climate change. Recent studies from around the world show very high resistance levels among nosocomial *A. baumannii* strains [19,20,21]. These factors indicate that community-acquired *A. baumannii* infections, caused by extensively drug-resistant strains, might become a common issue.

The antimicrobial resistance of the *A. baumannii* strains we studied coincides with global resistance trends. In our study, many *A. baumannii* strains were resistant to all tested antibiotics except colistin. *A. baumanni’s* multi-drug resistance significantly reduces patient treatment options [16]. Ampicillin and cefoperazone combinations with sulbactam, as well as colistin, appear to be the most effective drugs [22], as demonstrated when AMP-C-producing positive strains were tested for antibiotic resistance. A third of the *A. baumannii* strains tested positive for AMP-C production; this coincides with the other studies conducted in tertiary care institutions [23,24].

A novel approach to antibiotic-resistant bacteria is needed, as antimicrobial treatment options are running out for the cases caused by the most resistant bacteria strains. Various graphene derivatives have already been shown to have antimicrobial properties [25] and are being developed into products that will improve healthcare [26]. We have already tested the effects of the GO–Ag hybrid nanocomposite on *P. aeruginosa* [17]. The innate resistance mechanisms and acquired genomic variations [27] of *A. baumannii* might cause longer incubation times to be needed to significantly suspend the growth of bacteria colonies compared to *P. aeruginosa* [17]. Nevertheless, the GO–Ag hybrid nanocomposite was able to inhibit or reduce the growth of the tested *A. baumannii* strains. Resistance to cefepime, imipenem, meropenem, ciprofloxacin, doxycycline and tetracycline was associated with increased effectiveness of the GO–Ag hybrid nanocomposite against all *A. baumannii* strains. AMP-C production seems not to be a significant factor for the GO–Ag hybrid nanocomposite effect on its own. When the results were combined with antibiotic resistance, after 45 min of incubation, AMP-C-non-producing *A. baumannii* strains that are susceptible to cefepime, ceftazidime, imipenem, meropenem, piperacillin-tazobactam and tetracycline antibiotics were affected the least. Increasing the incubation time to 90 min significantly reduced the CFU numbers. Cefoperazone-sulbactam was the only case where resistance was associated with a significantly weaker inhibition of AMP-C-producing *A. baumannii* strains after 90 min of incubation.

The results showed that *A. baumannii* strains with lower antibiotic resistance levels were less affected after 45 min incubation with the GO–Ag hybrid nanocomposite than more resistant strains. Bacteria with strong resistance to certain antibiotics have been shown to be more susceptible to other antibiotics with different modes of action [28,29]. The difference between antibiotic-resistant and -susceptible strains’ survival also might be explained by how the GO–Ag hybrid nanocomposite interacts with bacteria cells [8,9]. The GO–Ag hybrid nanocomposite particles might block bacteria cells from interacting with the environment and exchanging essential nutrients, so the *A. baumannii* strains with higher metabolic needs (the ones producing antimicrobial neutralizing substances) might enter starvation sooner. Also, cells unable to excrete metabolites might become damaged due to osmotic pressure. The fact that prolonged incubation equalizes the effectiveness of the GO–Ag hybrid nanocomposite supports the theory that antibiotic susceptible strains are less affected due to lower metabolic needs.

## 5. Conclusions

A novel approach is needed to keep effectively fighting antibiotic-resistant *A. baumannii*, as well as other pathogens. Graphene-based materials are promising to be a prospective solution. Even though its effectiveness may vary due to various factors, prolonged pathogen–GO–Ag hybrid nanocomposite interaction helped to significantly reduce the vitality of most of the tested *A. baumannii* strains.

## Figures and Tables

**Table 1:** Comparison of antibiotic resistance levels among AMP-C-producing and AMP-C-non-producing *A. baumannii* strains.

Antibiotic	All *A. baumannii* Strains (*n* = 98) (*n* (%))	AMP-C -Producing (*n* = 32) (*n* (%))	AMP-C -Non-Producing (*n* = 66) (*n* (%))	*p*-Value
Ampicillin-sulbactam	75 (76.5)	13 (40.6)	62 (93.9)	<0.001
Cefoperazone-sulbactam	64 (65.3)	8 (25.0)	56 (84.8)	<0.001
Piperacillin-tazobactam	94 (95.5)	30 (93.8)	64 (97.0)	0.45
Cefepime	91 (92.9)	28 (87.5)	63 (95.5)	0.152
Ceftazidime	95 (96.9)	31 (96.9)	64 (97.0)	0.98
Imipenem	92 (93.9)	29 (90.6)	63 (95.5)	0.35
Meropenem	91 (92.9)	28 (87.5)	63 (95.5)	0.152
Gentamicin	82 (83.7)	22 (68.8)	60 (90.9)	0.005
Amikacin	81 (82.7)	22 (68.8)	59 (89.4)	0.011
Tobramycin	81 (82.7)	23 (71.9)	58 (87.9)	0.049
Ciprofloxacin	96 (98.0)	32 (100.0)	64 (97.0)	0.32
Doxycycline	69 (70.4)	21 (65.6)	48 (72.7)	0.47
Tetracycline	83 (84.7)	29 (90.6)	55 (83.3)	0.591
Tigecycline	69 (70.4)	18 (56.3)	51 (77.3)	0.033
Trimethoprim-sulfamethoxazole	86 (87.8)	31 (96.9)	55 (83.3)	0.055
Colistin	0 (0.0)	0 (0.0)	0 (0.0)	-

## Data Availability

Data not publicly shared due to ongoing research that includes this dataset.
